# Supplementary material for: A high-resolution mRNA expression time course of embryonic development in zebrafish
Source: eLife. 2017 Nov 16;6:e30860. doi: 10.7554/eLife.30860 (PMC5690287; doi:10.7554/eLife.30860)
Supplement: Supplementary file 6. [file elife-30860-supp6.zip › biolayout-clusters-files/Cluster104-genes.html]

Cluster104


# Cluster104: Genes

| Ensembl ID | Gene Name | Chr | Start | End | Biotype |
| --- | --- | --- | --- | --- | --- |
| ENSDARG00000087611 | ENSDARG00000087611 | 19 | 27244281 | 27260526 | protein\_coding |
| ENSDARG00000059083 | PPP2R5C | 17 | 1610625 | 1633474 | protein\_coding |
| ENSDARG00000027222 | adgrg1 | 7 | 28377526 | 28448137 | protein\_coding |
| ENSDARG00000080009 | bahcc1 | 12 | 34462111 | 34657411 | protein\_coding |
| ENSDARG00000041776 | ndst3 | 1 | 19453643 | 19580290 | protein\_coding |
| ENSDARG00000042069 | pebp1 | 5 | 14850737 | 14857602 | protein\_coding |
| ENSDARG00000076262 | pou3f2b | 16 | 32605791 | 32609062 | protein\_coding |
| ENSDARG00000028099 | sh2d3ca | 10 | 10252082 | 10320239 | protein\_coding |
| ENSDARG00000073944 | si:ch73-386h18.1 | 18 | 5898776 | 5942078 | protein\_coding |
| ENSDARG00000097368 | si:dkey-244a7.1 | 18 | 46255915 | 46260594 | protein\_coding |
| ENSDARG00000079125 | sowahab | 14 | 22150417 | 22157640 | protein\_coding |
| ENSDARG00000062338 | zeb2a | 9 | 25964947 | 26025606 | protein\_coding |
